# Computational insights into structural, electronic and optical characteristics of GeC/C_2_N van der Waals heterostructures: effects of strain engineering and electric field

**DOI:** 10.1039/c9ra08749d

**Published:** 2020-01-16

**Authors:** Hong T. T. Nguyen, Tuan V. Vu, Van Thinh Pham, Nguyen N. Hieu, Huynh V. Phuc, Bui D. Hoi, Nguyen T. T. Binh, M. Idrees, B. Amin, Chuong V. Nguyen

**Affiliations:** Division of Computational Physics, Institute for Computational Science, Ton Duc Thang University Ho Chi Minh City Vietnam nguyenthithamhong@tdtu.edu.vn; Faculty of Electrical & Electronics Engineering, Ton Duc Thang University Ho Chi Minh City Vietnam; Center of Excellence for Green Energy and Environmental Nanomaterials, Nguyen Tat Thanh University Ho Chi Minh City Vietnam; Institute of Research and Development, Duy Tan University Da Nang 550000 Vietnam nguyentthanhbinh8@duytan.edu.vn; Division of Theoretical Physics, Dong Thap University Cao Lanh 870000 Vietnam; Department of Physics, University of Education, Hue University Hue Vietnam; Department of Physics, Hazara University Mansehra 21300 Pakistan; Department of Physics, Abbottabad University of Science and Technology Abbottabad 22010 Pakistan; Department of Materials Science and Engineering, Le Quy Don Technical University Ha Noi 100000 Vietnam chuongnguyen11@gmail.com

## Abstract

Vertical heterostructures from two or more than two two-dimensional materials are recently considered as an effective tool for tuning the electronic properties of materials and for designing future high-performance nanodevices. Here, using first principles calculations, we propose a GeC/C_2_N van der Waals heterostructure and investigate its electronic and optical properties. We demonstrate that the intrinsic electronic properties of both GeC and C_2_N monolayers are quite preserved in GeC/C_2_N HTS owing to the weak forces. At the equilibrium configuration, GeC/C_2_N HTS forms the type-II band alignment with an indirect band gap of 0.42 eV, which can be considered to improve the effective separation of electrons and holes. Besides, GeC/C_2_N vdW-HTS exhibits strong absorption in both visible and near ultra-violet regions with an intensity of 10^5^ cm^−1^. The electronic properties of GeC/C_2_N HTS can be tuned by applying an electric field and vertical strains. The semiconductor to metal transition can be achieved in GeC/C_2_N HTS in the case when the positive electric field of +0.3 V Å^−1^ or the tensile vertical strain of −0.9 Å is applied. These findings demonstrate that GeC/C_2_N HTS can be used to design future high-performance multifunctional devices.

## Abstract 2

Vertical heterostructures from two or more than two two-dimensional materials are recently considered as an effective tool for tuning the electronic properties of materials and for designing future high-performance nanodevices.

## Graphical abstract

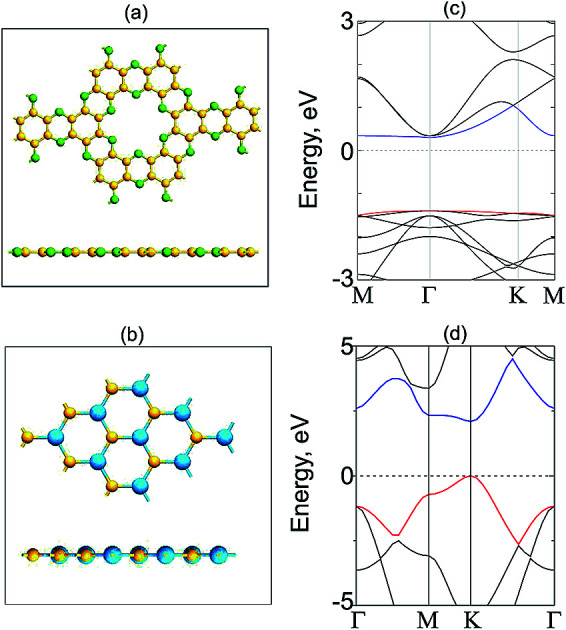

## 1. Introduction

Vertical heterostructures (HTSs) that are made layer-by-layer from two or more than two two-dimensional materials (2D) are recently considered as an effective tool for tuning the electronic properties of 2D materials and for designing future high-performance nanodevices owing to their promising properties, which may not be present in the individual 2D materials.^1^ The most common routes to synthesize the van der Waals (vdW) HTSs in experiments are chemical vapor deposition (CVD)^2,3^ and mechanical transfer process.^4,5^ To date, there are several vdW-HTSs based on 2D materials that have been experimentally fabricated, including graphene/transition metal dichalcogenides (TMDs),^6–8^ TMDs/TMDs,^9–11^ TMDs/BSe,^12^ TMDs/Mg(OH)_2_,^13^ and TMDs/BP^14^ which become promising candidates for future nanodevices, such as field-effect transistors (FETs), and tunnel diodes. Moreover, vdW-HTSs based on 2D materials have been proposed and investigated theoretically, such as GeSe/phosphorene,^15^ C_2_N/InSe,^16^ BP/MoSSe,^17^ arsenene/GaS,^18^ Ca(OH)_2_/arsenene,^19^ ZrS_2_/HfS_2_.^20^ All the above-mentioned studies demonstrate that constructing two 2D materials into vdW-HTSs provides an effective tool to design novel electronic and optoelectronic materials with favorable properties and novel phenomena, which are acceptable for future high-performance devices.

Recently, graphene-like GeC and C_2_N monolayers have been widely explored in different fields of optoelectronic and nanoelectronic applications owing to their extraordinary properties. For instance, the field-effect transistor based on C_2_N exhibits a high on/off ratio of 10^7^.^21^ Additionally, C_2_N monolayer shows extremely high selectivity and large permeance in favor of H_2_, which can be used for hydrogen separation.^22^ Monolayer C_2_N was obtained in experiments from a bottom-up wet–chemical reaction.^21^ Whereas, 2D GeC exhibits a dynamically stable planar structure and demonstrates excellent electronic and optical properties,^23,24^ high thermal conductivity,^25^ which make it potential material for electronic, optoelectronic, and photovoltaic devices.^26,27^ Moreover, it has been reported that GeC thin film can be synthesized by laser ablation,^28^ radio frequency reactive sputtering in Ar/CH_4_ (ref. 29) or plasma-enhanced CVD technique.^30^ Both GeC and C_2_N monolayers exhibit the semiconducting behavior with the direct band gap of about 2 eV.^21,23^ The electronic, transport and optical properties of GeC and C_2_N monolayer can be tuned by strains engineering,^31,32^ electric field,^33,34^ surface adsorption and functionalization.^35–37^ These properties make GeC and C_2_N materials to be suitable for the design of high-performance nanodevices.

More recently, the GeC-based and C_2_N-based vdW-HTSs have been experimentally fabricated and theoretically constructed, such as GeC/phosphorus,^38,39^ GeC/graphene,^40^ C_2_N/GaTe,^41^ C_2_N/graphene,^42^ C_2_N/TMDs,^43,44^ C_2_N/Sb^45^ and so forth. It is obvious that these vdW-HTSs preserve the intrinsic electronic properties of individual 2D materials and offer new opportunities for designing novel electronic and optoelectronic devices. For instance, Ren *et al.*^39^ reported that the excellent ability to capture visible light makes the blue-phosphorene/SiC vdW-HTS promising high-performance photocatalysts for water splitting. Wang *et al.*^42^ demonstrated that the C_2_N/Sb vdW-HTS has a tremendous opportunity to be applied in the photoelectronic device due to its tunable electronic properties under electric field and large power conversion efficiency. As far as we know, up to date, there is lack of the theoretical investigation on the structure and electronic properties of the combination between the GeC and C_2_N monolayers to form GeC/C_2_N vdW-HTS, as well as the effects of strain engineering and electric field. Therefore, in this work, we construct a novel GeC/C_2_N vdW-HTS and investigate its electronic properties using first-principles calculations. Moreover, the effects of vertical strains and electric field on the electronic properties of GeC/C_2_N vdW-HTS are also considered.

## 2. Computational details

In this work, the QUANTUM ESPRESSO simulated package,^46,47^ which is based on density functional theory (DFT) is used to perform all the geometric optimization and electronic properties calculations. The projected augmented wave was selected for describing the interaction between electron and ion for a plane-wave basis set within the cut-off energy of 510 eV. Whereas, in order to better describe the exchange-correlation energy, we adopted the generalized gradient approximation (GGA)^48^ within the Perdew–Burke–Ernzerhof (PBE) functional. Moreover, it is clear that traditional DFT approaches, including GGA–PBE are known to underestimate the band gap values of materials, but they can well predict the proper trend and physical mechanism. Indeed, to describe the weak forces, which are mainly dominated in layered materials, the dispersion corrected DFT-D3 is also used.^49^ All considered here materials are fully relaxed until energy and forces are converged to be 10^−6^ eV and 10^−3^ eV Å^−1^, respectively. A 9 × 9 × 1 and 6 × 6 × 1 Monkhorst–Pack *k*-point mesh in the Brillouin zone (BZ) was used in all our GGA–PBE and HSE calculations, respectively. To break the spurious interactions between the periodic surfaces, we applied a large vacuum thickness of 30 Å.

In addition, to check the structural stability of considered materials, we also calculate their binding energy and the phonon spectrum. The binding energy of considered heterostructures is calculated as follows:1
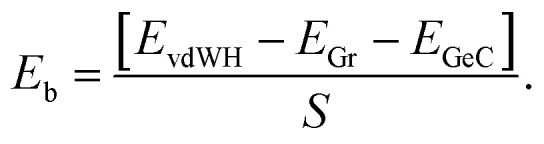
Here, *E*_vdWH_, *E*_Gr_ and *E*_GeC_, respectively, are the total energies of the constructing heterostructures, isolated Gr and GeC layers. *S* is the in-plane surface area of considered heterostructures.

The charge density difference in all considered here vdWHs can be obtained by:

2 Δ*ρ* = *ρ*_vdWH_ − *ρ*_Gr_ − *ρ*_GeC_,

where *ρ*_vdWH_, *ρ*_Gr_ and *ρ*_GeC_ are the charge densities of the considered vdWH, isolated Gr and GeC layers, respectively.

The optical absorption coefficient *α*(*ω*) of 2D systems can be obtained by:3
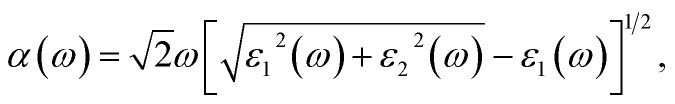
where *ε*_1_(*ω*) and *ε*_2_(*ω*) are the real and imaginary parts of the dielectric function, respectively.

## 3. Results and discussion

**Fig. 1:**
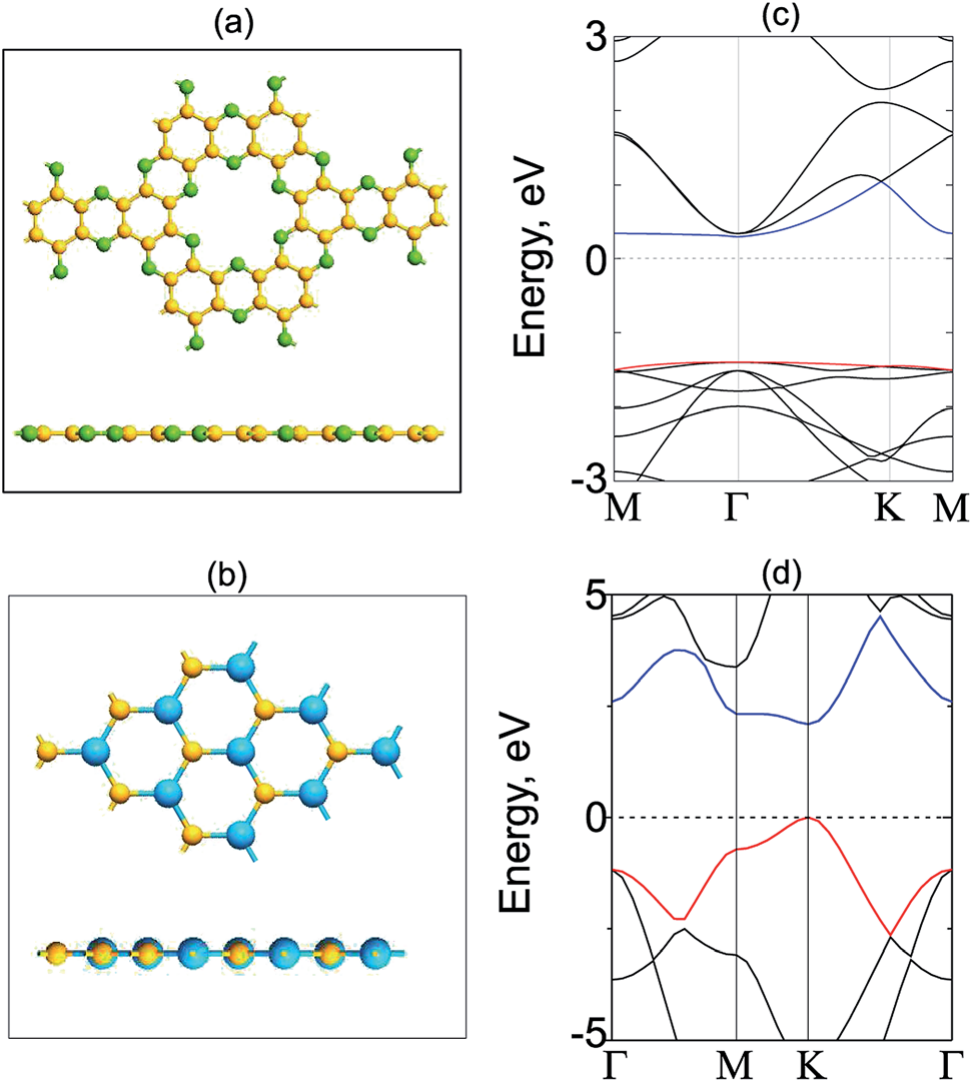
(a) and (b) are the atomic structures; (c) and (d) are the band structures of the C_2_N and GeC monolayers at the ground state. Yellow, green and blue balls stand for carbon, nitrogen and germanium atoms, respectively. Red and blue lines represent the valence band maximum (VBM) and conduction band minimum (CBM), respectively.

We first check the structural and electronic properties of the C_2_N and GeC monolayers at the ground state. The atomic structure of the C_2_N monolayer is displayed in Fig. 1(a), which indicates a planar honeycomb structure of C_2_ monolayer with benzene rings connected through nitrogen atoms. The calculated lattice parameter of the C_2_N monolayer is 8.33 Å and it is in agreement with previous experimental and theoretical reports.^21,50^ The C_2_N monolayer at the ground state exhibits a direct band gap semiconductor with both the valence band maximum (VBM) and conduction band minimum (CBM) at the M point, as shown in Fig. 1(c). The band gap of the C_2_N monolayer is calculated to be 1.74 eV. Similar to the atomic structures of the C_2_N monolayer, the GeC monolayer also displays a planar honeycomb structure. The lattice parameter and band gap of monolayer GeC are calculated to be 3.264 Å and 2.14 eV, respectively. These values are consistent with other reports.^23,51^ All the above-mentioned findings confirm that our used methods are reliable and they can be used to predict the correct trends in the physical properties of these materials.

**Fig. 2:**
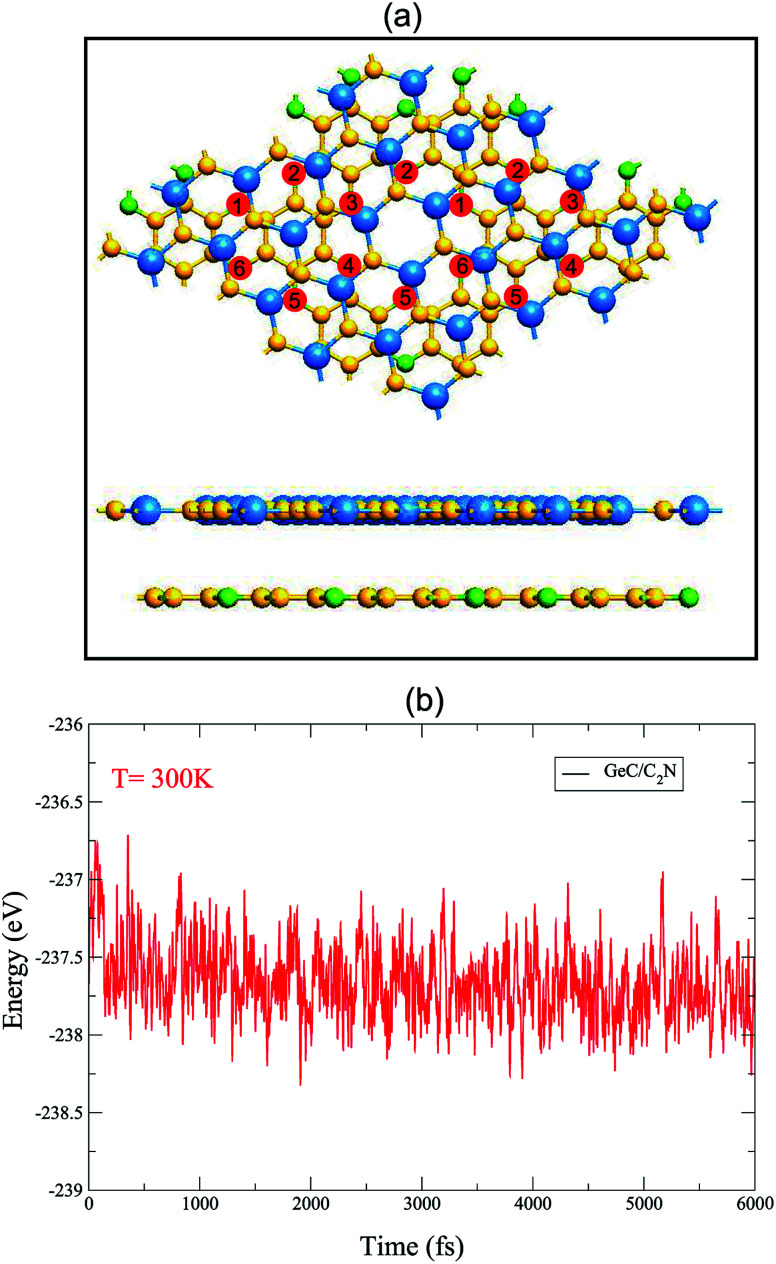
(a) Top view and side view of the relaxed atomic structure of GeC/C_2_N vdW-HTS. (b) *Ab initio* molecular dynamics calculation of the thermal stability of GeC/C_2_N vdW-HTS.

We now construct GeC/C_2_N vdW-HTS by vertically placing GeC on top of C_2_N layer, as depicted in Fig. 2(a). In order to minimize the lattice mismatch in the lattice parameter between GeC and C_2_N monolayers, we use a model of a supercell, containing a (2 × 2) C_2_N unit cell and (5 × 5). It is clear that the C_2_N monolayer is known to be a flexible structure and it can withstand strains 12%.^31^ The lattice constants of GeC, C_2_N supercells are 16.32 Å, 16.66 Å, respectively. We set the average of the lattice constants of GeC and C_2_N supercells (16.44 Å) as the lattice constant of the corresponding GeC/C_2_N vdW-HTS. The overall lattice mismatch of GeC/C_2_N vdW-HST is only 1.18%. As a result, the electronic properties of GeC/C_2_N vdW-HTS are still unchanged under such small strain. It should be noted that this lattice mismatch is very small and is consistent with that in previous reports.^52–56^ The atomic structure of GeC/C_2_N vdW-HTS after the structural optimization is depicted in Fig. 2(a). The interlayer distance, defining by *D*_eq_ between the GeC and C_2_N layers is obtained to be 3.43 Å. It is obvious that this *D*_eq_ is similar to that of other typical vdW heterostructures, such as GaN/BlueP,^57^ TMDs/GaN,^58^ graphene/GaS,^59^ ZnO/GeC,^60,61^ C_2_N/MX (M = Ga, In; X = S, Se, Te)^16,41,62^ and so forth. Such result demonstrates the typical vdW forces are mainly contributed in GeC/C_2_N HTS. Moreover, our calculated binding energy in such HTS is −79.34 meV Å^−2^, which is also comparable with that in other typical vdW-HSTs. Furthermore, in order to check the structural distortion and stability of GeC/C_2_N vdW-HTS, we perform *Ab initio* molecular dynamics, as depicted in Fig. 2(b). One can observe that GeC/C_2_N retains its geometric structure without any structural distortion after 6 ps. Moreover, one can find from Fig. 2(b) that the total energy fluctuation is small, indicating that GeC/C_2_N vdW-HTS is thermally stable.

**Fig. 3:**
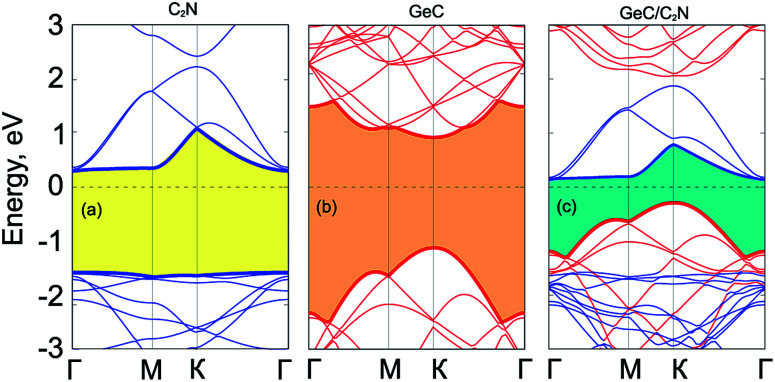
Electronic band structures of (a) monolayer C_2_N, (b) GeC and (c) GeC/C_2_N heterostructure. Red and blue lines represent the valence band maximum (VBM) and conduction band minimum (CBM), respectively.

Fig. 3 presents the electronic band structure of GeC/C_2_N vdW-HTS at the equilibrium state, along with that of the individual GeC and C_2_N monolayers. We can see that both the isolated GeC and C_2_N monolayers demonstrate the direct band gap semiconductors. When the GeC/C_2_N vdW-HTS is formed, it is obvious that its electronic band structure seems to be a combination between that of the individual GeC and C_2_N monolayers. This indicates the preservation of the intrinsic electronic properties of monolayers GeC and C_2_N in their GeC/C_2_N vdW-HTS. Moreover, we can observe that the band gap, forming in GeC/C_2_N vdW-HTS is significantly smaller than that of both GeC and C_2_N monolayers. The GeC/C_2_N vdW-HTS exhibits an indirect band gap semiconductor, which prevents the recombination of photoexcited electrons and holes, leading to a long carrier lifetime. As compared to the electronic band structures of GeC and C_2_N monolayers in Fig. 3(a) and (b), it can be seen that the VBM of such vdW-HTS is mainly contributed by the GeC layer, whereas the CBM of vdW-HTS comes from the CBM of C_2_N part, as displayed in Fig. 3(c). It also confirms the type-II band alignment, that is formed in such vdW-HTS. Interestingly, the type-II band alignment can be considered to improve the effective separation of electrons and holes. It demonstrates the advantage of such GeC/C_2_N vdW-HTS for designing optoelectronic devices that inhibit carrier recombination.

**Fig. 4:**
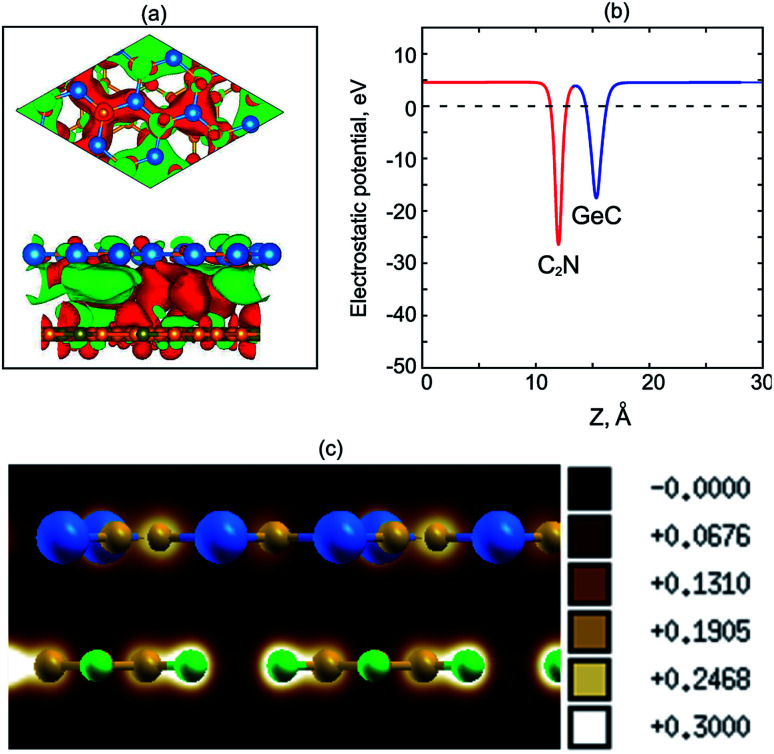
(a) Charge density difference and (b) electrostatic potential of GeC/C_2_N vdW-HTS. Red and green colors represent for the electron accumulation and depletion, respectively. (c) Electron localization functions of GeC/C_2_N vdW-HTS at the equilibrium state.

To have a clear picture of the charge transfer in GeC/C_2_N HST, we calculate its charge density difference, as shown in Fig. 4(a). We find that the charge densities distribution is mainly localized between the GeC and C_2_N layers. Moreover, one can observe that the electron is accumulated on the C_2_N layer and depleted on the GeC layer. The electron is likely transferred from the GeC to the C_2_N layers. The calculated work functions of isolated GeC and C_2_N monolayers are 4.91 eV and 5.68 eV, respectively, which confirm that the electrons are transported from the GeC to the C_2_N part in GeC/C_2_N vdW-HTS. This transportation results in a built-in electric field at the interface, leading to a reduction of the recombination of photogenerated electrons and holes. Fig. 4(b) shows the electrostatic potential of GeC/C_2_N HTS at the equilibrium state. We can see that the C_2_N layer has a deeper potential than the GeC layer. The difference in potential between the C_2_N and GeC layers is large of 13.4 eV at the equilibrium configuration, causing a charge transfer from the GeC to the C_2_N layers at the interface. Moreover, to confirm the existence of the vdW interactions between GeC and C_2_N monolayers, we further calculate the electron localization functions (ELFs) of GeC/C_2_N vdW-HTS, as depicted in Fig. 4(c). One can observe that there is no covalent bonding at the interfacial region of GeC/C_2_N vdW-HTS. From all of these findings, we can conclude that GeC/C_2_N vdW-HTS is featured *via* the weak vdW interactions.

**Fig. 5:**
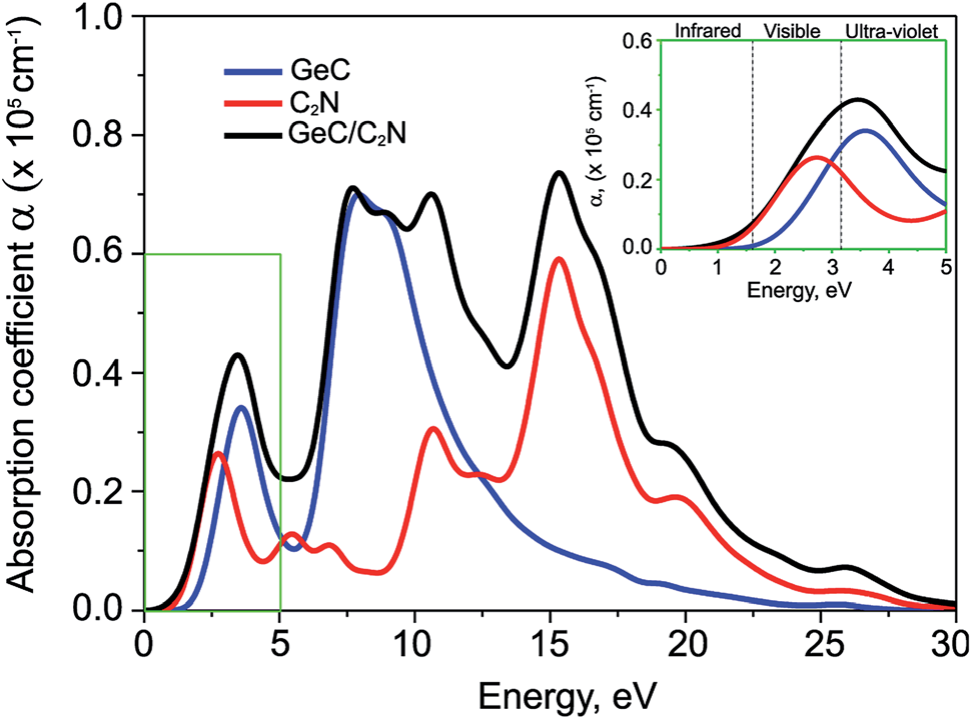
Calculated optical absorption coefficient of isolated GeC, C_2_N monolayers and their GeC/C_2_N vdW-HTS as a function of photon energy.

The optical absorption coefficient of GeC/C_2_N vdW-HTS is depicted in Fig. 5. In addition, the absorption coefficient of individual constituent GeC and C_2_N monolayers are also calculated for comparison. We find that the capacity of light adsorption of the GeC/C_2_N vdW-HTS is enhanced as compared to that of individual constituent monolayers. Thus, the GeC/C_2_N vdW-HTS exhibits excellent light-absorption ability. More interestingly, we find that GeC/C_2_N vdW-HTS exhibits strong absorption in both visible and near ultra-violet regions with an intensity of 10^5^ cm^−1^. It demonstrates that GeC/C_2_N vdW-HTS is an efficient material for photocatalysts and solar energy conversion.

**Fig. 6:**
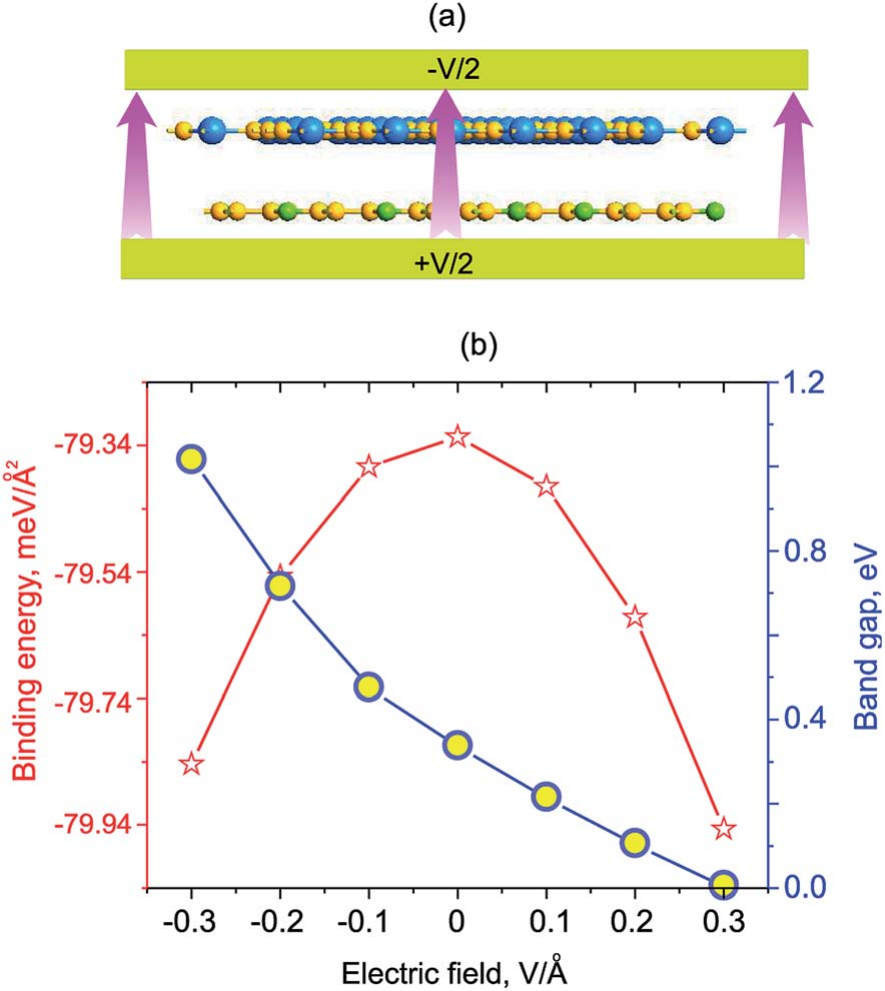
(a) Schematic model of the electric field, applying perpendicularly to the surface of GeC/C_2_N HTS. (b) Variation of the binding energy and band gap of GeC/C_2_N HTS as a function of the electric field.

Next, we will discuss the effects of electric field and strain engineering on the electronic properties of GeC/C_2_N vdW-HTS. The electric field applied perpendicularly along the *z* direction of the heterostructure, as illustrated in Fig. 6(a). The variation of the binding energy and band gap of GeC/C_2_N HTS under different strengths of electric fields is depicted in Fig. 6(b). We can see that applying an electric field tends to reduce the binding energy of the vdW-HTS. We find that the band gap of GeC/C_2_N vdW-HTS is very sensitive to the applied electric fields. The band gap depends not only on the strengths of the applied electric field, but also on its direction. When the positive electric field is applied, the band gap decreases with the increase of the positive electric field. Whereas, when the negative electric field is applied, the band gap increases with increasing the negative electric field. Interestingly, we find that GeC/C_2_N vdW-HTS changes from the semiconducting character to the metallic one when the positive electric field of +0.3 V Å^−1^ is applied. The semiconductor-to-metal transitions of GeC/C_2_N vdW-HTS make it a promising candidate for multifunctional nanodevices.

**Fig. 7:**
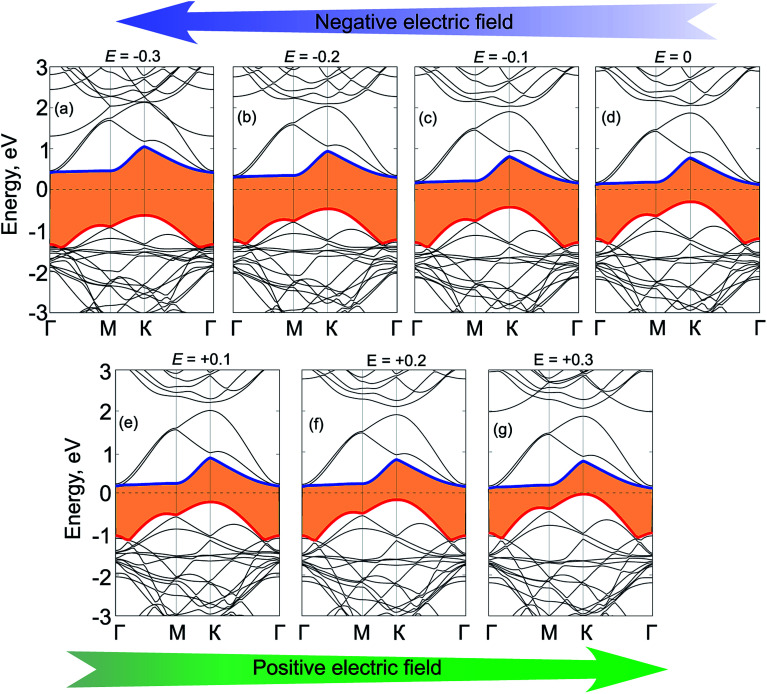
Band structures of GeC/C_2_N vdW-HTS under different strengths of electric fields, ranging from (a) −0.3 V Å^−1^, (b) −0.2 V Å^−1^, (c) −0.1 V Å^−1^, (d) 0 V Å^−1^, (e) +0.1 V Å^−1^, (f) +0.2 V Å^−1^, (g) +0.3 V Å^−1^.

To get further insights into the physical mechanism of the band gap of GeC/C_2_N HTS under electric fields, we further calculate its band structures under different strengths of the applied electric fields, as depicted in Fig. 7. We can see that applying a positive electric field tends to shift downwards the CBM, while the VBM is shifted upwards. This trend leads to a decrease in the band gap of such HTS. Under the critical strength of the positive electric field of +0.3 V Å^−1^, the VBM of HTS moves upwards and crosses the Fermi level, resulting in a transition from semiconductor to metal. The nature of such a decrease in the band gap of vdW-HTS is due to the reduction of the built-in electric field when the positive electric field is applied, which is opposite to that of the built-in electric field. On the other hand, the band gap of HTS is increased from 0.42 eV to 1.10 eV with decreasing the strengths of the negative electric field from 0 V Å^−1^ to −0.3 V Å^−1^, respectively. The type-II band alignment in GeC/C_2_N HTS, in this case, is still maintained with the CBM from the C_2_N layer and the VBM from GeC one.

**Fig. 8:**
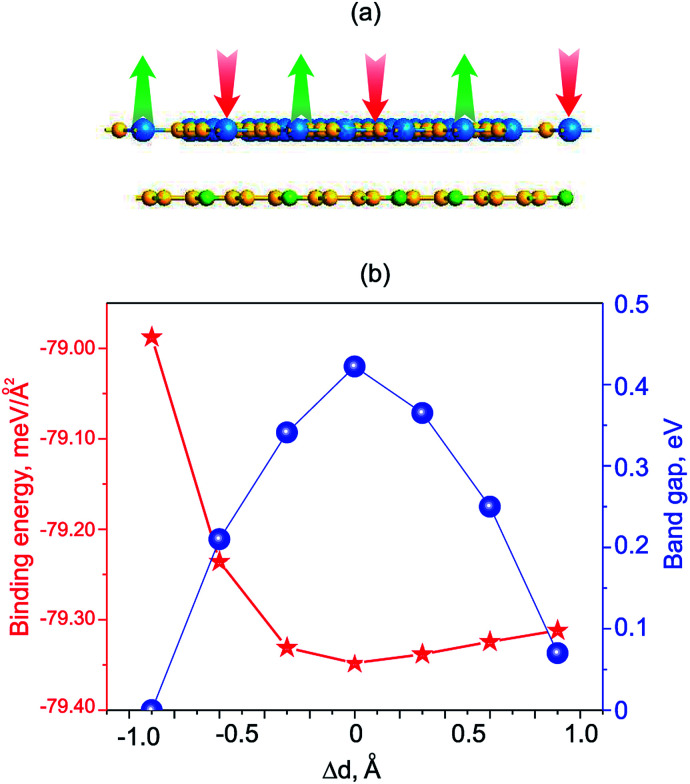
(a) Schematic model of GeC/C_2_N vdW-HTS under vertical strains. (b) Variation of the binding energy and band gap of GeC/C_2_N HTS as a function of strain engineering.

We now move to consider the case when the vertical strain is applied by changing the interlayer distance *d* between the GeC and C_2_N layers as follows: Δ*d* = *d*_0_ − *d*, where *d*_0_ and *d* is the strained and equilibrium (unstrained) interlayer distance. The schematic model of the vertical strain is depicted in Fig. 7(a). The change in the binding energy and band gap of GeC/C_2_N HTS is also calculated and plotted in Fig. 7(b). It is obvious that GeC/C_2_N HTS has the smallest binding energy at the equilibrium interlayer distance of 3.43 Å. The band gap of GeC/C_2_N HTS under vertical strains changes *via* two different ways, as illustrated in Fig. 8(b). When the tensile strain is applied, *i.e.* Δ*d* > 0, the band gap of vdW-HTS decreases from 0.42 eV to approximately 0 eV with increasing Δ*d* from 0 Å to +0.9 Å, respectively. On the contrary, the band gap decreases from 0.42 eV to 0 eV with the decrease of the compressive strain from Δ*d* = 0 Å to Δ*d* = −0.9 Å, respectively. The physical mechanism of pressure-induced band gap narrowing is related to the positions of the VBM and CBM of GeC/C_2_N vdW-HTS under compressive strain. One can observe that the compressive strain has little effect on the vdW-HTS. Moreover, the band gaps narrowing when the interlayer distance is decreased can be explained by the fact that the compressive strain cannot facilitate electron transfer from the GeC to the C_2_N layer and the hole transfer from C_2_N to the GeC layer. Thus, the VBM upshifts towards the Fermi level, thus the band gap of the GeC/C_2_N vdW-HTS narrows. Also, it is obvious that the band gap of GeC/C_2_N vdW-HTS is more sensitive to the compressive strain than the tensile strain. However, as we can see from Fig. 8(b), both the compressive and tensile strains can cause the transition from semiconductor to the metal in GeC/C_2_N vdW-HTS.

**Fig. 9:**
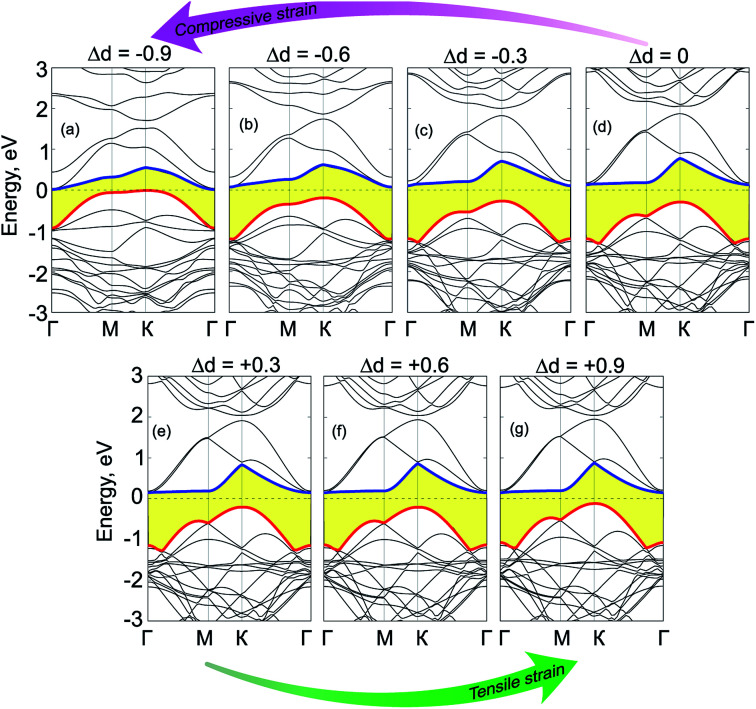
Band structures of GeC/C_2_N vdW-HTS under different strengths of electric fields, ranging from (a) Δ*d* = −0.9 Å, (b) Δ*d* = −0.6 Å, (c) Δ*d* = −0.3 Å, (d) Δ*d* = 0 Å, (e) Δ*d* = + 0.3 Å, (f) Δ*d* = + 0.6 Å, (g) Δ*d* = + 0.9 Å.

Fig. 9 shows the electronic band structures of GeC/C_2_N vdW-HTS under compressive and tensile vertical strains. When the compressive strain is applied, *i.e.* Δ*d* < 0, we can observe that both the VBM and CBM of such vdW-HTS move towards the Fermi level, leading to a decrease in the band gap. When a large compressive strain of Δ*d* = −0.9 Å, both the CBM and VBM of GeC/C_2_N HTS cross the Fermi level, forming the metallic feature of such vdW-HTS. Similar to the compressive strain, the tensile strain also tends to shift the VBM and CBM of GeC/C_2_N vdW-HTS towards the Fermi level. This trend leads to a decrease in the band gap of the vdW-HTS and may cause the transition from semiconductor to metal under a large strain. Therefore, we can conclude that the GeC/C_2_N vdW-HTS is known to have an indirect band gap semiconductor and to feature the type-II band alignment, facilitating the effective separation of photogenerated electrons and holes. This is advantageous for fabricating optoelectronic devices that inhibit carrier recombination. Moreover, the optical absorption of GeC/C_2_N vdW-HTS is enhanced in both visible and near UV light as compared with that of the individual constituent monolayers, making it a promising candidate for light-absorption applications and solar energy conversion.

## 4. Conclusion

In summary, we have designed GeC/C_2_N vdW-HTS and systematically investigated its structure and electronic properties through first principles calculations. Our results show that GeC/C_2_N vdW-HTS is mainly characterized by the weak vdW interaction, leading to the preservation of the electronic features of both GeC and C_2_N monolayers. At the equilibrium state with the interlayer distance of 3.43 Å and the binding energy of −79.34 meV Å^−2^, we find that GeC/C_2_N HTS has a semiconductor with an indirect band gap of 0.42 eV, which is slightly smaller than that of the individual GeC and C_2_N monolayers. Moreover, GeC/C_2_N displays the type-II band alignment, confirming its ability for designing optoelectronic devices that inhibit carrier recombination. Besides, GeC/C_2_N vdW-HTS exhibits strong absorption in both visible and near-ultraviolet regions and its capacity of light adsorption is enhanced as compared to that of individual constituent monolayers. Furthermore, both the electric field and vertical strain can effectively tune the electronic properties of GeC/C_2_N vdW-HTS. The semiconductor to metal transition can emerge in GeC/C_2_N vdW-HTS when the positive electric field of +0.3 V Å^−1^ or the tensile vertical strain of −0.9 Å is applied. All these findings make GeC/C_2_N a promising candidate for future optoelectronic and nanoelectronic devices.

## Conflicts of interest

There are no conflicts to declare.

## Supplementary Material
